# Gut Microbiota Diversity and Function in Adults With Type 2 Diabetes, Alzheimer’s Disease, and Both Conditions

**DOI:** 10.1155/ijm/5247744

**Published:** 2026-04-05

**Authors:** Alexis M. Rodríguez-Rosas, Diana L. Baldenebro-Félix, Jesús M. Peréz-Villarreal, Jaime García-Mena, Carlos A. Kawano-Soto, Josué Camberos-Barraza, Marco A. Valdéz-Flores, Loranda Calderón-Zamora, Carla E. Angulo-Rojo, Javier A. Magaña-Gómez, Alma M. Guadrón-Llanos

**Affiliations:** ^1^ Programa de Posgrado en Ciencias Biomédicas, Facultad de Ciencias Químico-Biológicas, Universidad Autónoma de Sinaloa, Culiacán, Sinaloa, Mexico, uas.edu.mx; ^2^ Laboratorio de Neurociencias, Facultad de Medicina, Universidad Autónoma de Sinaloa, Culiacán, Sinaloa, Mexico, uas.edu.mx; ^3^ Departamento de Genética y Biología Molecular, Cinvestav, Ciudad de Mexico, Mexico, cinvestav.mx; ^4^ Clínica de Geriatría, Hospital Regional Dr. Manuel Cárdenas de la Vega ISSSTE, Culiacán, Sinaloa, Mexico; ^5^ Facultad de Medicina, Universidad Autónoma de Sinaloa, Culiacán, Sinaloa, Mexico, uas.edu.mx; ^6^ Facultad de Biología, Universidad Autónoma de Sinaloa, Culiacán, Sinaloa, Mexico, uas.edu.mx; ^7^ Laboratorio de Nutrición Molecular, Facultad de Ciencias de la Nutrición y Gastronomía, Universidad Autónoma de Sinaloa, Culiacán, Sinaloa, Mexico, uas.edu.mx; ^8^ Laboratorio de Diabetes y Comorbilidades, Facultad de Medicina, Universidad Autónoma de Sinaloa, Culiacán, Sinaloa, Mexico, uas.edu.mx

## Abstract

**Introduction:**

Type 2 diabetes mellitus (T2DM) is known to increase the risk of Alzheimer’s disease (AD), but the role of the gut microbiota in this relationship is not fully understood. This study investigated the gut microbiota profiles of adults with T2DM, adults with AD, both conditions (AD–T2DM), and healthy controls to identify patterns associated with metabolic and neurodegenerative conditions.

**Methods:**

A cross‐sectional study was conducted with 148 participants divided into six groups: CTRL < 60 years, CTRL ≥ 60 years, T2DM < 60 years, T2DM ≥ 60 years, AD, and AD–T2DM. Clinical assessments and 16S rRNA gene sequencing of fecal samples were performed to analyze microbial diversity and composition.

**Results:**

Compared with controls, older adults with T2DM, AD, and AD–T2DM presented reduced microbial diversity and distinct microbial compositions. Notably, SCFA‐producing genera (*Veillonella* and *Dialister*) decreased in T2DM patients ≥ 60 years, whereas *Roseburia* and *Blautia* were more abundant in AD patients and those with *AD–T2DM*. GDP‐mannose biosynthesis was downregulated in AD–T2DM patients.

**Conclusion:**

This study highlights changes in the microbiota in T2DM and AD–T2DM patients, suggesting that targeting these microbial alterations could offer new prevention strategies for metabolic‐neurodegenerative comorbidities.

**Significance Statement:**

T2DM and AD share metabolic and inflammatory pathways, yet their combined impact on the gut microbiota remains unexplored. By profiling 148 adults, healthy individuals, those with T2DM, AD, and co‐occurring AD–T2DM, using 16S rRNA gene V4 region sequencing, we identified specific dysbiosis in AD–T2DM. Rather than examining T2DM and AD as independent conditions, the present study conceptualizes their coexistence as a metabolic–neurodegenerative interaction state and assesses whether this comorbidity is associated with a distinct gut microbiota composition and predicted functional profile. Therefore, we examine the gut microbiota of adults with AD–T2DM using an age‐stratified design in an underrepresented Mexican population, integrating clinical, metabolic, cognitive, taxonomic, and functional data to explore potential mechanisms underlying metabolic–neurodegenerative crosstalk. These findings identify microbial genera that may mediate the “diabeto‐neuro” crosstalk, offering novel targets for early intervention and precision microbiota‐based therapies to mitigate metabolic‐neurodegenerative comorbidity.

## 1. Introduction

Type 2 diabetes mellitus (T2DM) is a chronic metabolic disorder characterized by insulin resistance, impaired pancreatic β‐cell function, and resultant hyperglycemia [1]. T2DM is associated with dysregulated glucose transport and metabolism, including alterations in the expression and function of glucose transporter (GLUT) proteins, which mediate facilitated diffusion of glucose across cellular membranes in insulin‐sensitive tissues [2]. In recent decades, T2DM has emerged as a global epidemic associated not only with cardiovascular and renal complications but also with an increased risk of developing Alzheimer’s disease (AD) [3, 4]. Epidemiological and molecular evidence show that chronic hyperglycemia and insulin resistance promote the cerebral accumulation of β‐amyloid peptide and the hyperphosphorylation of tau protein, the two main pathological hallmarks of AD [5, 6]. Moreover, the limited efficacy of current treatments and the lack of diagnostic tools capable of detecting AD in preclinical stages underscore the need to identify new biomarkers and deepen the understanding of the molecular mechanisms linking both disorders [7, 8]. The gut–brain axis has garnered increasing attention, as the gut microbiota modulates various metabolic, immune, and neuroinflammatory processes through the production of metabolites such as short‐chain fatty acids (SCFAs), lipopolysaccharides (LPS), and trimethylamine N‐oxide; these compounds influence both intestinal permeability and glial activation [9–11]. While gut‐derived metabolites such as SCFA and LPS are recognized mediators of the gut–brain axis, the present study explores these mechanisms indirectly through taxonomic profiling and predictive functional analyses rather than direct metabolite quantification. Imbalances in microbial composition, observed in both patients with T2DM and those with AD, appear to amplify chronic neuroinflammation and brain insulin dysfunction, thereby strengthening the hypothesis that AD is a “brain metabolic disorder” [12, 13].

Although gut microbiota alterations have been independently described in T2DM and AD, considerably less is known about the microbial landscape in individuals affected by both conditions. The AD–T2DM comorbidity represents a clinically relevant but understudied phenotype in which metabolic dysfunction and neurodegeneration converge. Given this context, it is essential to characterize the gut microbiota associated with both pathologies comprehensively [13]. To our knowledge, this study is among the first to examine alterations in gut microbiota in an age‐stratified cohort comprising T2DM, AD, and AD–T2DM groups within a Mexican population. By integrating clinical, cognitive, taxonomic, and predicted functional analyses, we aim to delineate microbial signatures associated with each condition and explore whether dysbiosis patterns reflect shared metabolic aging processes or disease‐specific features. Our objective is to describe the gut microbiota profile in individuals with T2DM and AD, identify patterns of dysbiosis and altered metabolic pathways, and support the development of targeted microbiota‐based interventions to slow or prevent neurodegenerative progression before irreversible clinical manifestations.

## 2. Materials and Methods

### 2.1. Study Design and Study Population

An observational, cross‐sectional, and comparative study was conducted. The participants were divided into four stratified groups: (1) healthy controls (CTRL), comprising 60 participants; (2) T2DM, comprising 60 participants; (3) AD, comprising 17 participants; and (4) AD–T2DM, comprising 11 participants. Additionally, the CTRL (CTRL < 60 years: 30 participants, CTRL ≥ 60 years: 30 participants) and T2DM (T2DM < 60 years: 30 participants, T2DM ≥ 60 years: 30 participants) groups were subdivided by age into subgroups of 30–59 years and ≥ 60 years. The diagnosis of AD or AD–T2DM was based on a prior clinical evaluation by a board‐certified neurologist. The diagnostic criteria included a specialist‐administered cognitive assessment battery combined with neuroimaging studies (computed tomography), in accordance with standard clinical practice. Although formal staging of AD severity (e.g., mild, moderate, or advanced stage) was not available, global cognitive performance was assessed in all participants using the Montreal cognitive assessment (MoCA). Participants who had received systemic antibiotic treatment within the three months prior to stool sample collection were excluded to minimize antibiotic‐related alterations in gut microbiota composition. The groups were formed through open invitations to the general population and recruitment at the ISSSTE General Hospital “Dr. Manuel Cardenas de la Vega” in Culiacan, Mexico. Before inclusion, all subjects received detailed verbal and written information about the study procedures and provided informed consent following the principles outlined in the Declaration of Helsinki. Ethical approval was granted by the Ethics Committee of the Faculty of Medicine at the Autonomous University of Sinaloa (registration number CEI‐FM‐PI‐2022‐010).

### 2.2. Clinical and Anthropometric Data Collection

A clinical history was obtained from each participant. Anthropometric measurements followed the standards of the International Society for the Advancement of Kinanthropometry [14]. Height was measured with a SECA 213 stadiometer, and body weight was measured using a Tanita BC‐545 N electronic scale; both measurements were taken under fasting conditions and with light clothing. Body mass index (BMI) was calculated in kg/m^2^ [15]. Peripheral blood samples were collected after 12 h of fasting to quantify glucose levels; lipid profiles, including triglycerides, total cholesterol, high‐density lipoprotein cholesterol (HDL‐C), low‐density lipoprotein cholesterol (LDL‐C), and very low‐density lipoprotein cholesterol (VLDL‐C) and glycated hemoglobin (HbA1c) levels were all measured from these blood samples.

### 2.3. Cognitive and Psychological Evaluation

Cognitive status was assessed using the MoCA, which is designed to distinguish between mild cognitive impairment and AD, for score correction in subjects with low educational levels [16]. Sleep quality was evaluated with the Athens Insomnia Scale [17], whereas the severity of depressive symptoms was determined via the 21‐item Beck Depression Inventory (BDI) [18, 19]. The participants were classified based on the obtained scores as follows: 0 to 13, indicating no depression; 14 to 19, mild depression; 20 to 28, moderate depression; and 29 to 63, severe depression [19].

### 2.4. Collection and Processing of Stool Samples

Each participant provided a stool sample collected in a sterile container and preserved at −80°C until processing. Genomic DNA was isolated from 200 mg of fecal material via the ZymoBIOMICS DNA Miniprep Kit (Zymo Research Corp., Irvine, CA, USA) following the manufacturer’s protocol. The purity of the isolated DNA was verified by the A260/280 and A260/230 absorbance ratios via a NanoDrop 2000 (Thermo Scientific). Integrity was assessed by 1% agarose gel electrophoresis.

### 2.5. Amplification and Sequencing of the V4 Region of the 16S rRNA Gene

The V4 region of the bacterial 16S rRNA gene was amplified by endpoint PCR using the forward in‐house designed primer iSeq V4‐520F (5′TCG TCG GCA GCG TCA GAT GTG TAT AAG AGA CAG‐AYT GGG YDT AAA GNG‐3′), where the first 33 bases were taken from the “16S amplicon PCR forward primer” [20] and the last 15 italicized bases complementary to coordinates 562–576 of the *Escherichia coli* 16S rDNA molecule rrnB GenBank J01859.1 [21] were taken from Table S2 of a systematic study of microbial diversity investigations using 16S rRNA amplicon genes [22]. The reverse primer was the 16S amplicon PCR reverse primer (5ʹ‐GTC TCG TGG GCT CGG AGA TGT GTA TAA GAG ACA G‐GAC TAC HVG GGT ATC TAA TCC‐3ʹ), where the last 15 italicized bases are complementary to coordinates 784–805 of the *Escherichia coli* 16S rDNA molecule rrnB GenBank J01859.1 [21]. These primers generated a 311 bp amplicon. The PCR program was 95°C for 3 min, 25 cycles of (95°C for 30 s, 55°C for 30 s, and 72°C for 30 s), and 72°C for 3 min. Following purification with AMPure XP (Beckman Coulter, Brea, CA, USA), a second indexing PCR (8 cycles) was performed via the Illumina Nextera XT Index v2 kit to allow multiplexing of the samples. Libraries were quantified on a Qubit 2.0 (Invitrogen), and the size distribution was verified on a Bioanalyzer 2100 (Agilent), yielding fragments of 388 bp. Finally, the libraries were normalized to a final concentration of 1 nM, with the BIOMICS control serving as an internal standard, and the samples were sequenced on an Illumina iSeq 100 platform (2 × 150 bp).

### 2.6. Statistical and Bioinformatics Analysis

Anthropometric, biochemical, and cognitive variables were summarized as median with interquartile range (IQR). Comparisons between groups were performed using the Kruskal–Wallis test, followed by Dunn’s multiple comparisons test when appropriate. All descriptive and inferential statistical analyses were conducted using GraphPad Prism 9, with a *p* value < 0.05 as statistically significant.

Raw paired‐end reads were processed using QIIME2 (v2024.2). Low‐quality sequences and primers were removed, and amplicon sequence variants (ASVs) were inferred using DADA2 with chimera filtering. Taxonomy was assigned using a naïve Bayes classifier trained on the Greengenes database (Version 13_8). Analyses were performed in R (v4.2.1) with the *phyloseq* package (v1.40.0) to calculate relative abundances [23], generate rarefaction curves (Supporting information, Figure S1), and alpha diversity metrics (Observed, Chao1, Shannon, and Simpson). Beta diversity was assessed using weighted UniFrac distances and visualized through nonmetric multidimensional scaling (NMDS). The NMDS model was visualized using the Shepard diagram to verify the adjustment of the analysis (Supporting information, Figure S3). Differential abundance was tested with *DESeq2* (v1.36.0), adjusting *p*‐values with the Benjamini–Hochberg for false discovery rate (FDR). Multivariable microbiome association analysis was performed using the MaAsLin2 package. ASV relative abundance data and clinical metadata were used as input. The primary model included study group, age, sex, HbA1c, visceral fat, HDL cholesterol, creatinine, antibiotic exposure within the past 3 months, stool consistency category, cognitive status (MoCA category), and depression status (BDI category) as fixed effects. Microbial features were filtered using a minimum prevalence threshold of 10% and a minimum relative abundance of 0.001. Data were normalized using total sum scaling and log‐transformed prior to analysis. Associations were tested using linear models with covariate standardization. Finally, functional metagenomic prediction with PICRUSt2 and pathway differences were analyzed in STAMP with FDR < 0.05 [24, 25].

## 3. Results

### 3.1. Participant Characteristics

The analysis of anthropometric and biochemical characteristics summarized in Table 1 revealed that, in the subgroup < 60 years, individuals with T2DM had a significantly greater BMI and visceral fat percentage than the controls did, along with a persistent reduction in HDL‐C levels. Furthermore, increased concentrations of VLDL‐C and triglycerides were detected, reflecting a pro‐atherogenic lipid profile. In subgroup ≥ 60 years, although differences in anthropometric parameters were less evident, subjects with T2DM maintained lower HDL‐C levels and higher triglyceride and VLDL‐C levels than did those in the CTRL ≥ 60 years and AD groups, without statistically significant variations in total cholesterol or LDL‐C concentrations.

**TABLE 1:** Age, anthropometric, and biochemical characteristics of the groups studied.

Variable/group	CTRL < 60 years	T2DM < 60 years	CTRL ≥ 60 years	T2DM ≥ 60 years	AD	AD–T2DM	*p*
Age (years)	44.00 (33.75–57.00)	52.00 (49.00–55.00)	66.50 (62.75–69.00)	66.00 (64.00–70.00)	80.00 (71.50–82.50)	84.00 (74.00–90.00)	< 0.0001^b,c,d,e,f,g,h,i^
Weight (kg)	68.50 (60.30–85.30)	86.10 (71.93–97.73)	66.90 (60.40–74.30)	76.80 (67.58–83.33)^z^	60.10 (45.20–76.25)	66.10 (61.40–77.90)	< 0.0001^a,f,h,m^
Height (cm)	161.00 (154.40–168.00)	165.9 (158.40–169.90)	157.90 (153.10–163.20)	157.70 (152.80–162.60)	152.50 (148.00–162.50)	153.00 (146.30–170.00)	0.001^h^
BMI (kg/m^2^)	26.40 (24.43–30.33)	30.10 (27.05–34.43)	26.20 (25.08–29.28)	29.00 (25.98–33.05)	23.50 (21.20–29.80)	29.40 (26.70–32.80)	< 0.0001^a,f,h^
Waist–hip ratio (WHR)	0.81 (0.78–0.86)	0.89 (0.85–0.98)	0.84 (0.77–0.88)	0.90 (0.84–0.95)	0.87 (0.77–0.99)	0.92 (0.86–0.97)	NS
Visceral fat (%)	7.75 (5.37–9.50)	11.50 (8.87–15.00)	10.00 (8.87–13.63)	12.50 (10.00–16.50)	11.50 (7.50–16.50)	12.50 (10.13–15.75)	< 0.0001^a,b,c,d,e^
Total cholesterol (mg/dL)	174.00 (154.50–196.00)	191.00 (168.00–214.00)	191.00 (154.5–224.00)	164.50 (138.30–205.50)	166.20 (131.80–194.80)	190.80 (138.00–226.10)	NS
HDL‐C (mg/dL)	52.00 (41.75–62.25)	43.00 (34.50–53.00)	53.00 (44.75–59.00)	40.50 (33.75–48.50)	49.05 (43.33–60.65)	41.70 (37.30–51.40)	0.0002^c,j,m^
LDL‐C (mg/dL)	99.80 (84.35–138.1)	115.80 (93.60–130.30)	119.10 (82.55–144.60)	86.20 (64.75–133.90)	95.63 (68.57–122.30)	166.90 (101.20–212.20)	NS
VLDL‐C (mg/dL)	19.60 (14.45–26.50)	35.00 (25.50–42.80)	24.60 (14.60–29.70)	31.90 (24.30–42.00)	17.81 (14.20–22.21)	20.08 (16.54–28.90)	< 0.0001^a,c,f,h,i,m^
Triglycerides (mg/dL)	98.00 (72.25–132.50)	175.00 (127.00–214.00)	128.5 (73.00–150.80)	159.50 (120.00–210.00)	89.06 (71.00–111.00)	100.40 (82.70–144.50)	< 0.0001^a,c,f,h,i,m^
Glucose (mg/dL)	87.00 (82.00–94.00)	139.90 (110.50–188.50)	89.00 (83.75–94.50)	102.50 (92.00–130.30)	84.10 (80.18–90.48)	131.40 (91.30–150.90)	< 0.0001^a,c,e,f,h,j,l,m,o^
HbA1c (%)	5.65 (5.27–6.02)	7.22 (5.96–8.63)	5.70 (5.30–5.90)	6.80 (5.80–7.25)	5.25 (5.02–5.75)	7.00 (6.00–8.10)	< 0.0001^a,c,e,f,h,j,l,m,o^

*Note:* Values are presented as medians with interquartile range (IQR). Variables following a normal distribution (weight, BMI, total cholesterol, and HDL‐C) were compared using one‐way ANOVA with Tukey’s post hoc test for multiple comparisons. Variables deviating from normality (age, height, WHR, visceral fat, VLDL‐C, LDL‐C, triglycerides, glucose, and HbA1c) were analyzed by the Kruskal–Wallis test with Dunn’s post hoc correction. Superscripts ^a,b,c,d,e,f,g,h,i,j,l,m,n,o^ denote significant pairwise differences (*p* < 0.05) between groups (^a^: CTRL < 60 years vs. T2DM < 60 years; ^b^: CTRL < 60 years vs. CTRL ≥ 60 years; ^c^: CTRL < 60 years vs. T2DM ≥ 60 years; ^d^: CTRL < 60 years vs. AD; ^e^: CTRL < 60 years vs. AD–T2DM; ^f^: T2DM < 60 years vs. CTRL ≥ 60 years; ^g^: T2DM < 60 years vs. T2DM ≥ 60 years; ^h^: T2DM < 60 years vs. AD; ^i^: T2DM < 60 years vs. AD–T2DM; ^j^: CTRL ≥ 60 years vs. T2DM ≥ 60 years; ^l^: CTRL ≥ 60 years vs. AD–T2DM; ^m^: T2DM ≥ 60 years vs. AD; ^o^: AD vs. AD–T2DM); NS indicates nonsignificant comparisons (*p* ≥ 0.05).

In the MoCA score, participants with AD (with or without T2DM) obtained significantly lower scores compared to the other groups (*p* < 0.001), confirming the cognitive impairment characteristic of this pathology. For depressive symptomatology, as assessed by the BDI, AD patients presented significantly higher scores than the remaining groups (*p* < 0.001), whereas the other groups showed low and relatively homogeneous values. Regarding sleep quality, measured by the Athens Insomnia Scale, no statistically significant differences were detected between the groups (Table 2).

**TABLE 2:** Psychological‐cognitive evaluation of the groups studied.

Variable/Group	CTRL < 60 years	T2DM < 60 years	CTRL ≥ 60 years	T2DM ≥ 60 years	AD	AD–T2DM	*p*
Montreal cognitive assessment (MoCA)	25.00 (23.00–27.00)	24.00 (21.00–25.00)	23.00 (21.00–25.00)	23.00 (21–25.25)	9.00 (3.00–14.50)	10.00 (4.00–13.00)	< 0.0001^d,e,h,i,k,l,m,n^
Beck Depression Inventory (BDI)	4.50 (2.00–6.75)	6.50 (3.00–11.50)	4.00 (2.50–7.25)	5.00 (1.00–7.00)	10.00 (6.00–22.00)	9.00 (6.00–18.00)	0.0007^d,k,m^
Athens Insomnia Scale (AIS)	3.00 (2.00–6.00)	4.50 (2.00–7.25)	2.50 (1.00–6.25)	4.00 (0.75–7.00)	6.00 (2.00–11.00)	4.00 (3.00–11.00)	NS

*Note:* Data are presented as median with interquartile range (IQR). Between‐group differences for MoCA and BDI scores (normally distributed) were assessed by one‐way ANOVA followed by Tukey’s multiple‐comparison test; AIS scores (non‐normal distribution) were compared using the Kruskal–Wallis test with Dunn’s post hoc correction. Superscripts ^d,e,h,I,k,l,m,n^ denote significant pairwise differences (*p* < 0.05) between groups (^d^: CTRL < 60 years vs. AD; ^e^: CTRL < 60 years vs. AD–T2DM; ^h^: T2DM < 60 years vs. AD; ^i^: T2DM < 60 years vs. AD–T2DM; ^k^: CTRL ≥ 60 years vs. AD; ^l^: CTRL ≥ 60 years vs. AD–T2DM; ^m^: T2DM ≥ 60 years vs. AD; ^n^: T2DM ≥ 60 years vs. AD–T2DM); NS indicates nonsignificant comparisons (*p* ≥ 0.05).

### 3.2. Relative Abundance and Core Microbiota

In the phylogenetic characterization of the gut microbiota (Supporting information, Figure S2), relative abundance analysis at the phylum level revealed that Firmicutes_A and Bacteroidota were the dominant groups in the bacterial community, representing 65%–70% and 15%–20%, respectively, of all the groups evaluated. Although no statistically significant differences were found at this taxonomic level, the data suggest slight variations associated with the presence of T2DM and AD.

At the genus level, more than 60% of the ASVs were identified; the remainder were grouped as “Others” because each did not reach at least 1% relative abundance in any study group, reflecting both the low prevalence and the resolution limits of the V4 region of the 16S rRNA gene (Figure 1(a)). In both the T2DM ≥ 60 years group and the CTR ≥ 60 years group, *Blautia* was the most abundant genus, whereas *Prevotella* was enriched in the AD group. In the heatmap (Figure 1(b)), genera were hierarchically organized according to their abundance profiles and associated with their corresponding phyla, facilitating the visualization of shared patterns and differences between groups.

FIGURE 1Distribution of the percentages of microbial genera in each study group. (a) Stacked bars represent the relative abundance of different genera, reflecting variations in the overall composition of the gut microbiota according to the clinical condition of each group. (b) Heatmap of the core microbiota of microbial genera in each of the study groups with a cutoff point of genera present in at least 40% of the samples. The rows represent the different genera, organized via a hierarchical clustering dendrogram, while the columns group the samples in each group. The color scale reflects the level of abundance, and the box on the left indicates the phylum to which each genus belongs.

**Figure:**
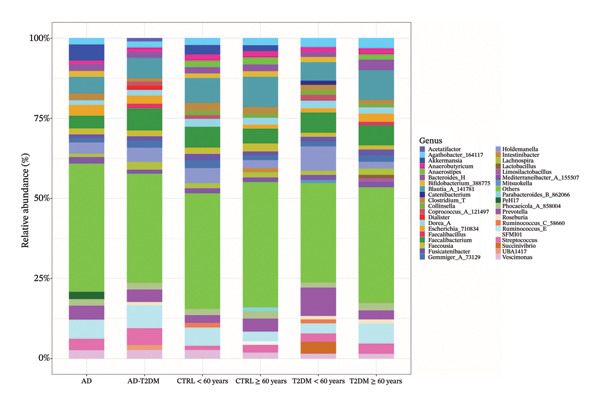
(a)

**Figure:**
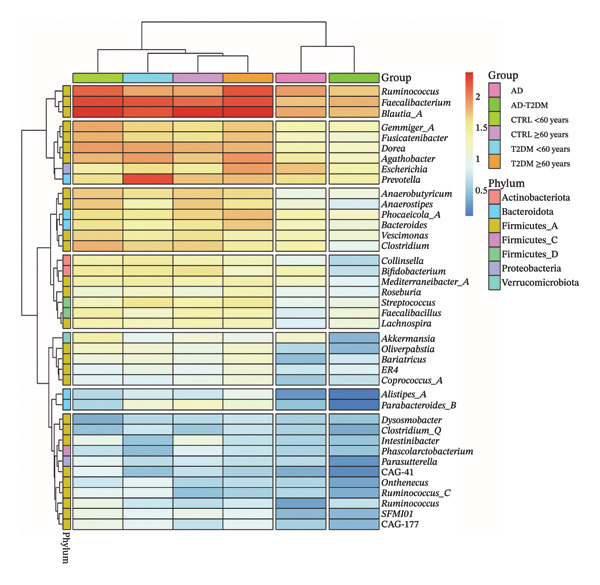
(b)

### 3.3. Alpha and Beta Diversity of the Gut Microbiota Among the Groups

**FIGURE 2:**
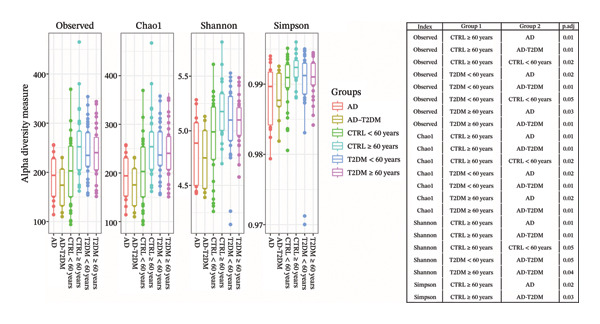
Comparison of alpha diversity metrics (observed, Chao1, Shannon, and Simpson indices) among the study groups. Each panel shows boxplots with the median and spread of values in each group (CTRL < 60 years [*n* = 30], T2DM < 60 years [*n* = 30], CTRL ≥ 60 years [*n* = 30], T2DM ≥ 60 years [*n* = 30], AD [*n* = 17], and AD–T2DM [*n* = 11]), illustrating differences in microbial community richness and evenness according to clinical condition. Statistical comparisons between groups were performed via two‐sided Wilcoxon rank‐sum tests for each index (observed, Chao1, Shannon, and Simpson), with test statistics (W) reported and *p* values adjusted for multiple testing via the Benjamini–Hochberg (BH) procedure. The table alongside the plot reports the significant BH‐adjusted *p* values for pairwise comparison.

Pairwise comparisons of alpha diversity metrics across all study groups (Figure 2) demonstrated clear age‐ and disease‐associated declines in microbial richness and evenness. In participants aged ≥ 60 years, both observed and Chao1 richness were significantly lower in the CTRL ≥ 60 versus CTRL < 60 (observed FDR = 0.02; Chao1 FDR = 0.02), CTRL ≥ 60 versus AD (observed FDR = 0.01; Chao1 FDR = 0.01), and CTRL ≥ 60 versus AD–T2DM (observed FDR = 0.01; Chao1 FDR = 0.01) groups. Similarly, compared with those with T2DM < 60, those with AD–T2DM presented reduced observed and Chao1 richness (observed FDR = 0.01; Chao1 FDR = 0.01). Shannon diversity was also significantly lower in the CTRL ≥ 60 versus AD (FDR = 0.01) and versus AD–T2DM (FDR = 0.01) groups, and Simpson evenness declined in the CTRL ≥ 60 versus AD (FDR = 0.05) and versus AD–T2DM (FDR = 0.03) groups. A notable reduction in Simpson evenness further distinguished T2DM ≥ 60 from CTRL ≥ 60 (FDR = 0.05). A significant reduction in observed richness was found in T2DM < 60 versus CTRL < 60 (FDR = 0.05), whereas the Shannon, Chao1, and Simpson indices remained comparable. In contrast, no significant differences were detected in the observed, Chao1, Shannon, or Simpson indices among the CTRL < 60, T2DM < 60, AD, and AD–T2DM subgroups in the younger age groups.

**FIGURE 3:**
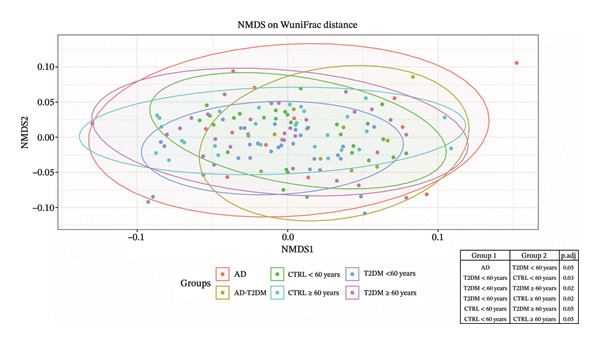
NMDS ordination of the gut microbiota composition on the basis of weighted UniFrac distances. Nonmetric multidimensional scaling (NMDS) was performed on rarefied 16S rRNA gene profiles via weighted UniFrac distances to compare the community structure across six study groups: AD (red), CTRL < 60 years (green), T2DM < 60 years (blue), AD–T2DM (gold), CTRL ≥ 60 years (teal), and T2DM ≥ 60 years (pink). Each point represents an individual sample; shaded ellipses indicate 95% confidence intervals around the group centroids. The distinct clustering along NMDS1 and NMDS2 highlights both age‐related and disease‐specific shifts in intestinal microbial communities. NMDS of weighted UniFrac distances was used to visualize overall community differences among the study groups, followed by ANOSIM to test for global separation. Pairwise ANOSIM comparisons were then performed across all unique group combinations via the same distance metric. The resulting *p* values were adjusted for multiple testing via the Benjamini–Hochberg (BH) procedure. The table alongside the plot reports the significant BH‐adjusted *p* values for pairwise comparison.

In pairwise comparisons of beta diversity‐based weighted UniFrac distances (Figure 3), the T2DM < 60‐year subgroup differed significantly from both the CTRL < 60‐year subgroup (FDR = 0.03) and the CTRL ≥ 60‐year subgroup (FDR = 0.02), which showed reduced similarity to the T2DM ≥ 60‐year subgroup (FDR = 0.02) and the AD subgroup, with significant divergence (FDR = 0.05). CTRL < 60 years also diverged from the CTRL ≥ 60 years (FDR = 0.05) and T2DM ≥ 60 years (FDR = 0.05), reflecting expected microbiota changes with age. Consequently, while the overall community structure appears broadly similar, we cannot exclude finer‐scale shifts at the species or strain level or more subtle functional differences that might emerge with deeper sequencing.

### 3.4. Differential Abundance Analysis

Using DESeq2, the most relevant changes in the abundance of bacterial genera between the different study groups were identified. Differential abundance analysis revealed distinct shifts in key gut bacterial genera across age and disease strata (Figure 4). In the comparison between CTRL < 60 years versus older controls (CTRL ≥ 60 years; panel A), *Holdemanella* was markedly depleted in elderly individuals, whereas enrichments of *Veillonella*, *Prevotella,* and *Streptococcus* were observed. In contrast, older individuals with T2DM (T2DM ≥ 60 years) presented significant overrepresentation of *Lactobacillus* and *Streptococcus* relative to young controls (panel B), accompanied by a reciprocal loss of *Holdemanella*. Comparison of the AD group with the young control group (panel C) revealed that *Eubacterium* and *Erysipelatoclostridium* were enriched in the control group. Finally, in the AD–T2DM comorbidity (panel D), multiple taxa, such as *Anaerostipes, Dialister*, *Catenibacterium,* and *Bifidobacterium,* were significantly increased, whereas *Streptococcus* was reduced relative to young controls. In subjects aged 30–59 years (CTRL < 60 years vs. T2DM < 60 years), significant enrichment of genera such as *Veillonella_A*, *Blautia*, *Ruminococcus*, and *Lactobacillus* was observed in the T2DM < 60 years group (Figure 5 panel A). Moreover, *Prevotella* and *Coprococcus* were more abundant in the CTRL < 60 years group. When comparing older adults with and without diabetes (CTRL ≥ 60 years vs. T2DM ≥ 60 years), a decrease in *Veillonella*, *Dialister*, and *Prevotella* was observed in T2DM ≥ 60 years, counterbalanced by a notable increase in *Lactobacillus*, *Ruminococcus C_58660*, and *Faecalibacterium*.

FIGURE 4Differential abundance of gut microbiota genera across age and disease cohorts determined via DESeq2. The log_2_ fold‐change values for each genus are shown for (a) CTRL ≥ 60 years versus CTRL < 60 years, (b) CTRL < 60 years versus T2DM ≥ 60 years, (c) CTRL < 60 years versus AD, (d) CTRL < 60 years versus AD–T2DM, (e) CTRL < 60 years versus T2DM < 60 years, (f) CTRL ≥ 60 years versus T2DM ≥ 60 years, (g) CTRL ≥ 60 years versus AD, and (h) CTRL ≥ 60 years versus AD–T2DM. Normalization was conducted via size factors obtained from the geometric means of the counts, and the Wald test was employed to calculate differences between groups. The false discovery rate (FDR) was used to adjust the *p* values. Changes in log_2_ abundance are represented by horizontal bars, and bacterial taxa with q values < 0.05 are labeled along the *Y*‐axis.

**Figure:**
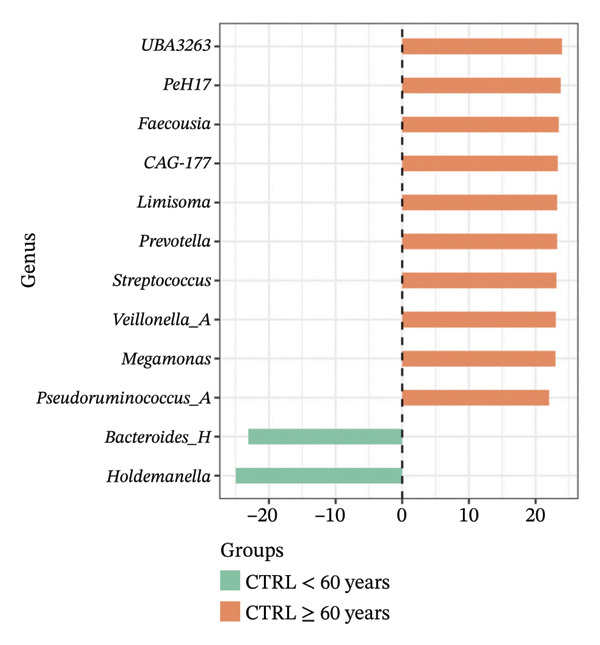
(a)

**Figure:**
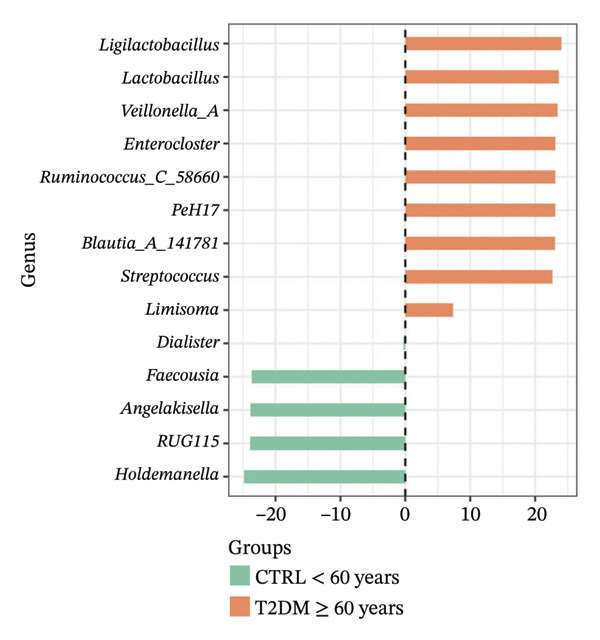
(b)

**Figure:**
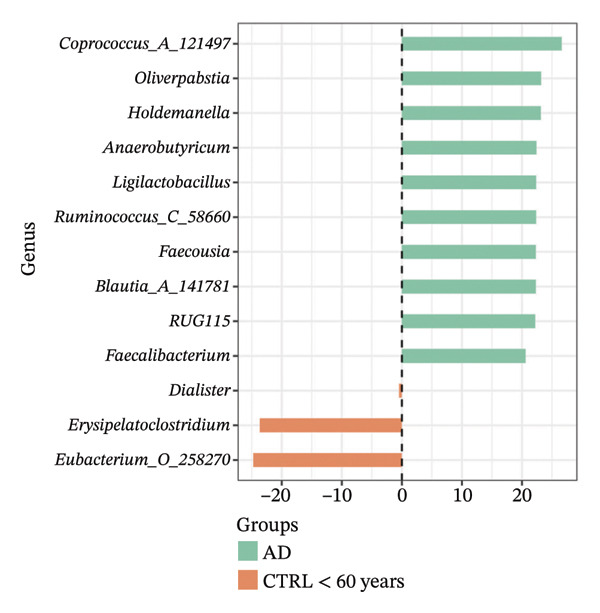
(c)

**Figure:**
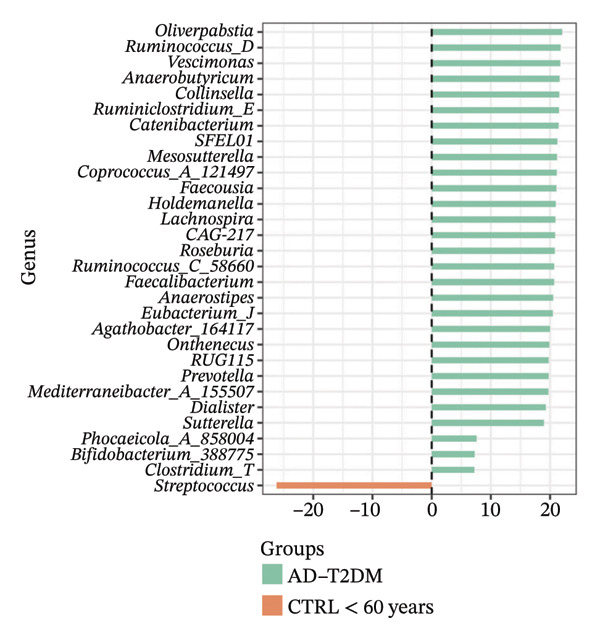
(d)

**Figure:**
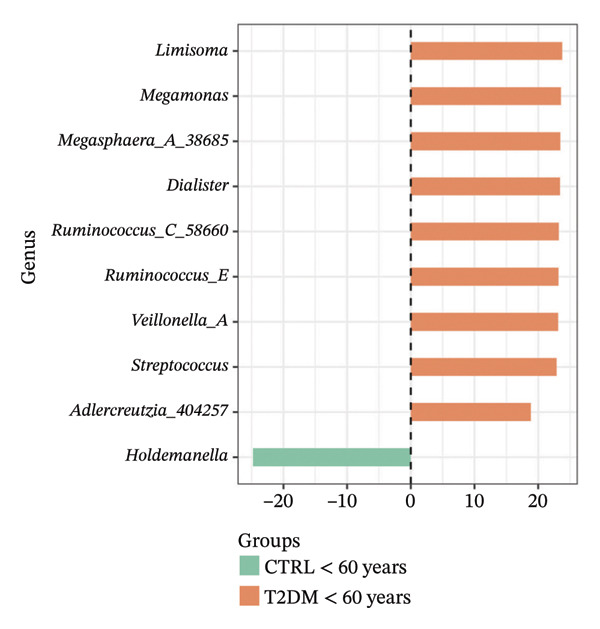
(e)

**Figure:**
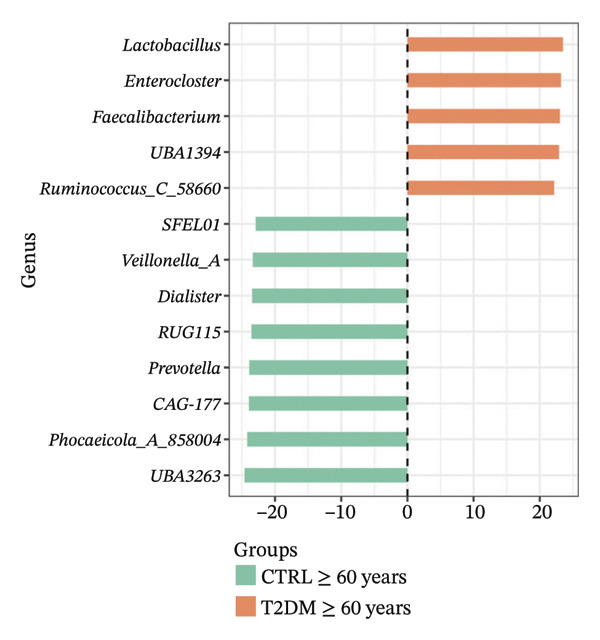
(f)

**Figure:**
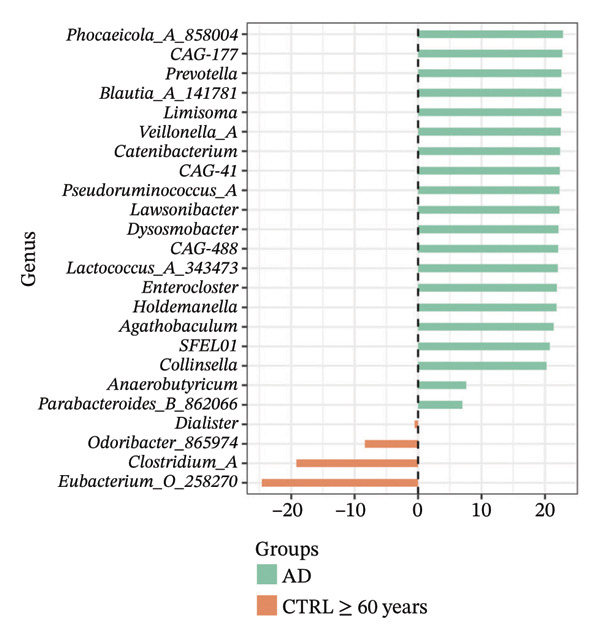
(g)

**Figure:**
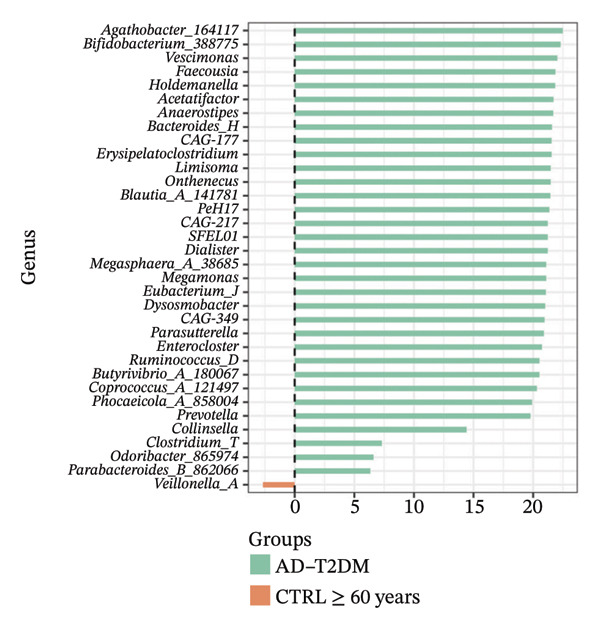
(h)

FIGURE 5Multivariable associations between microbial features and study groups identified using MaAsLin2. Boxplots show relative abundance distributions of microbial features significantly associated with study groups after multivariable adjustment. Features were filtered using a minimum prevalence threshold of 10% and a minimum relative abundance of 0.001, normalized by total sum scaling, and log‐transformed prior to modeling. Statistical significance was determined using Benjamini–Hochberg FDR correction, with FDR < 0.05 considered significant. (a) Boxplot showing the relative abundance of a *Prevotella*‐assigned amplicon sequence variant significantly enriched in participants with T2DM < 60 years (FDR = 6.84 × 10^−3^). (b) Boxplot showing a second independent *Prevotella*‐assigned amplicon sequence variant also significantly associated with T2DM < 60 years (FDR = 1.79 × 10^−2^). Boxes represent the median and interquartile range (IQR); whiskers indicate 1.5 × IQR; individual points correspond to single samples.

**Figure:**
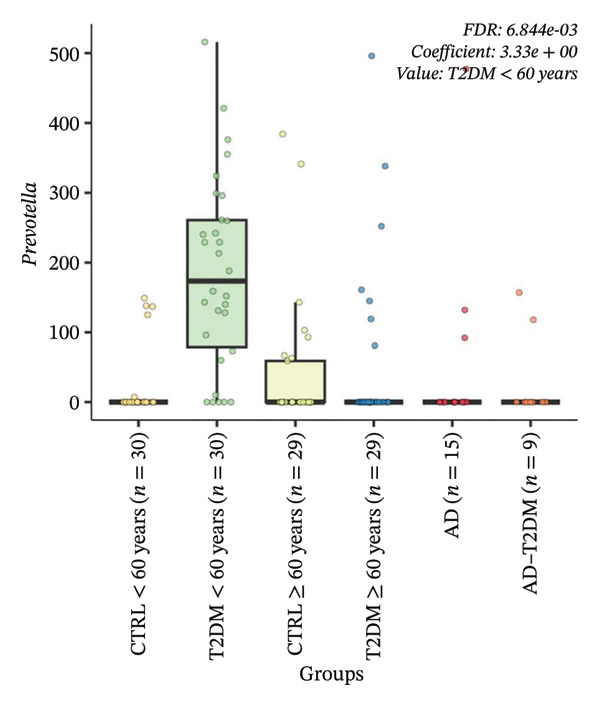
(a)

**Figure:**
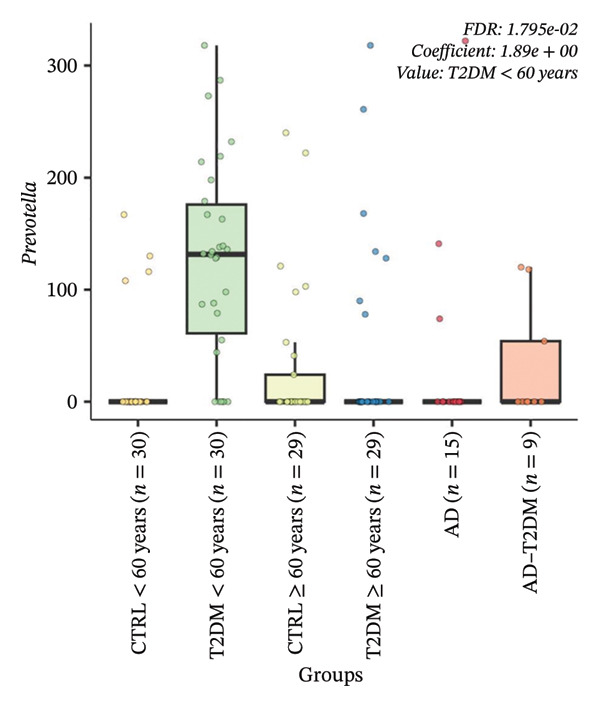
(b)

A comparison between the CTRL ≥ 60 years and AD groups revealed a greater relative abundance of the genera *Lactococcus*, *Blautia*, *Roseburia,* and *Limisoma*, among others, in the AD group. Moreover, *Eubacterium* and *Odoribacter* were reduced. When comorbidity (AD–T2DM vs. CTRL ≥ 60 years) was considered, an enrichment of *Vescimonas*, *Megamonas*, *Blautia*, *Holdemanella*, *Bacteroides_H,* and *Anaerostipes* was confirmed.

### 3.5. Exploration of Associations Between the Gut Microbiota and Clinical Parameters

Multivariable association analysis identified four microbial features significantly associated with the study group after covariate adjustment and FDR correction (Supporting information, Table S1). Two independent features taxonomically assigned to the genus *Prevotella* were significantly enriched in participants with T2DM > 60 years, showing positive regression coefficients ranging from 1.89 to 3.33 and FDR values between 0.006 and 0.017 (Figure 5). No microbial features remained significantly associated with CTRL, AD, AD–T2DM, or older T2DM groups after multivariable adjustment. These findings define a distinct multivariable‐adjusted microbial signature associated with younger T2DM status.

### 3.6. Functional Metagenomic Prediction

Functional analysis of PICRUSt2 revealed variations in multiple metabolic pathways, particularly those related to amino acids and lipids. However, after applying the FDR correction, only the GDP‐mannose biosynthesis pathway in the AD–T2DM group remained significantly different from the CTRL ≥ 60‐year group (Supporting information, Figure S4). This suggests that this change may reflect specific microbial adaptations in older individuals with diabetes. Moreover, the remaining analyzed pathways did not meet the threshold for statistical reliability.

## 4. Discussion

The increasing prevalence of both T2DM and AD in the aging population has prompted numerous studies exploring their potential epidemiological and biological links. Compared with normoglycemia, T2DM is associated with up to a 50% increased risk of developing dementia, including AD [6, 26, 27]. Given that AD‐related neuropathological changes begin years before clinical symptoms emerge, identifying early biomarkers in individuals with T2DM that signal an increased risk of neurodegeneration is crucial [27]. Gut microbiota profiles have emerged as promising indicators for elucidating the underlying neuroinflammatory mechanisms [13].

Our anthropometric data revealed that participants with T2DM had significantly higher BMIs and waist circumferences than metabolically healthy controls. Central obesity, a hallmark of T2DM, is also a risk factor for late‐life cognitive decline, partly due to insulin resistance and chronic low‐grade inflammation associated with excess adiposity [28]. These findings align with previous research linking increased adiposity to greater brain atrophy and poorer cognitive performance [29].

Consistent with the anthropometric profile, participants with T2DM also presented elevated fasting blood glucose and HbA1c levels, indicators of suboptimal glycemic control [30]. These chronic metabolic imbalances are relevant to neurodegeneration, as persistent hyperglycemia promotes the formation of advanced glycation end products (AGEs), which accumulate in endothelial and neural tissues, contributing to oxidative stress and neuroinflammation [31, 32]. Moreover, AGEs can exacerbate β‐amyloid aggregation and tau hyperphosphorylation, key neuropathological events in AD [32, 33].

Depressive symptoms, assessed using the BDI, were more prevalent in participants with AD, while individuals with T2DM showed generally low and relatively homogeneous scores, with a tendency toward higher values compared with controls, a concern since depression independently raises dementia risk and may worsen diabetes‐related cognitive decline [34]. At the molecular level, chronic depression in diabetes, linked to elevated proinflammatory cytokines and cortisol, can intensify neuroinflammation and synaptic dysfunction, suggesting T2DM alone heightens vulnerability to cognitive and emotional disturbances [35].

A central focus of this study was the potential role of the gut microbiota in mediating the relationship between metabolic dysfunction and neuroinflammation in T2DM [13]. Alpha diversity analysis revealed a decrease in bacterial richness and diversity in the T2DM < 60 years and T2DM ≥ 60 years groups compared with the healthy controls. A similar trend was evident in the AD and AD–T2DM groups. This loss of microbial diversity is consistent with prior studies on chronic metabolic conditions, where reduced diversity is associated with Western diets and chronic inflammation [36, 37]. Such low diversity may reflect the loss of beneficial bacteria and a less resilient gut ecosystem [38]. Beta diversity analysis further confirmed distinct microbial community structures in the T2DM, AD, and AD–T2DM groups compared with healthy controls. These findings align with evidence that metabolic and neurodegenerative disorders share altered gut microbiota profiles [39–42]. However, it is important to note that gut microbiota composition is strongly influenced by geographic region, dietary habits, and ethnic background, which may account for variability in the specific taxa reported across studies [43, 44]. For example, decreases in genera such as *Faecalibacterium* and *Roseburia* have been reported in some AD cohorts, whereas other studies have reported preserved or even increased abundance depending on dietary and regional factors [45–47]. While reduced diversity appears to be a consistent feature, the direction and magnitude of changes in individual genera have differed across cohorts worldwide [37, 40, 48, 49]. In this context, the present findings from a Mexican population contribute to a growing body of evidence highlighting both shared and population‐specific features of gut dysbiosis associated with T2DM and AD, underscoring the importance of epidemiological and geographic context when interpreting microbiome alterations.

Notably, age‐associated microbial shifts, as observed in the present study, have also been reported in other metabolic disorders linked to cognitive decline, including obesity and insulin resistance [50, 51]. Across these conditions, aging has been associated with reduced gut microbial diversity and compositional changes affecting SCFA‐producing and barrier‐protective taxa, as well as an enrichment of inflammation‐associated genera [36, 38]. Such patterns have been described as part of a broader metabolic aging phenotype, characterized by chronic low‐grade inflammation, impaired gut barrier integrity, and altered host–microbe metabolic interactions [52]. In this context, our findings suggest that part of the observed dysbiosis may reflect shared metabolic aging processes rather than disease‐exclusive microbial signatures.

At the taxonomic level, these broader metabolic aging‐associated patterns are reflected in specific, biologically meaningful shifts in individual bacterial genera across age and disease strata in our cohort. Taxonomic analysis identified microbial patterns with potential pathogenic relevance. These shifts mostly match with trends reported for aging and metabolic dysfunction, although a few observations merit further consideration [52]. The marked depletion of *Holdemanella* in older versus younger controls (panel A) aligns with evidence that many butyrate‐producing Firmicutes decline with age [53–55]. Similarly, the enrichment of *Streptococcus* and *Veillonella* in elderly individuals echoes prior reports of increased opportunistic taxa in seniors [56–58]. Furthermore, the overrepresentation of *Lactobacillus* and *Streptococcus* in older T2DM patients (panel B) has also been documented in diabetic cohorts, reflecting hyperglycemia‐driven substrate availability and metformin‐mediated intestinal acidification [59, 60]. The reciprocal loss of *Holdemanella* highlights the potential compounding of age‐ and diabetes‐related dysbiosis, which could exacerbate gut barrier disruption and systemic inflammation [61, 62].

Older adults with T2DM showed depleted gram‐positive, SCFA‐producing genera (e.g., *Prevotella*, *Holdemanella*, and *Dialister*), likely reducing butyrate, propionate, and acetate levels that maintain gut barrier integrity and reduce inflammation [63–69]. Such losses not only exacerbate metabolic inflammation but also may have downstream consequences for neurodegenerative processes: Preclinical models have demonstrated that depletion of SCFA‐producing bacteria may be detrimental to AD pathogenesis [70]. Furthermore, decreased SCFA levels are associated with decreased expression of tight junction proteins such as ZO‐1 and occludin [71, 72], promoting LPS translocation and systemic endotoxemia [73, 74]. This inflammatory state may impair insulin signaling by promoting the phosphorylation of insulin receptor substrate 1, inhibiting the PI3K/Akt pathway, and reducing GLUT4 translocation [73]. Notably, most participants with T2DM were being treated with metformin, a drug that affects both glycemia and the composition of the gut microbiota [75]. This therapy could modulate the proliferation of genera such as *Lactobacillus* and consequently influence inflammatory and metabolic pathways, decreasing the expression of tight junction proteins and increasing permeability, facilitating endotoxemia and systemic inflammation [76, 77]. Future research should control for medication type, dose, and duration to disentangle treatment from disease effects.

Interestingly, the microbial profile in the AD and AD–T2DM groups showed a divergent pattern from previously reported findings. Whereas prior studies have noted reductions in butyrate‐producing genera, such as *Roseburia*, *Faecalibacterium*, and *Anaerostipes*, and increased *Proteobacteria* in AD, our data revealed elevated levels of *Roseburia*, *Anaerostipes*, *Blautia*, *Prevotella*, *Parabacteroides*, *Lactococcus*, and *Bifidobacterium* [78, 79]. These genera are typically associated with efficient fiber fermentation and SCFA production, suggesting that regional dietary, genetic, or lifestyle factors may uniquely modulate the gut microbiota in our population [80]. For example, the high abundance of *Prevotella* may reflect fiber‐rich diets with greater consumption of complex carbohydrates and vegetables [81]. Similarly, elevated *Collinsella*, a genus linked to inflammation, in AD patients could indicate compromised gut barrier function and LPS‐mediated endotoxemia [82]. Although increased relative abundance of *Proteobacteria* has been reported in some AD cohorts and interpreted as a marker of gut dysbiosis and inflammation, we did not observe this increase in our study population. Recent evidence further indicates that *Proteobacteria* levels may be highly context‐dependent and responsive to microbiome modulation, as probiotic intervention has been shown to reduce *Proteobacteria* abundance in AD models [83]. Together, these findings highlight the heterogeneity of microbiota alterations in AD across populations and study designs.

In the AD–T2DM group, we observed elevated levels of T2DM‐associated genera, such as *Bacteroides* and *Blautia*, which coexist with typically beneficial genera. This was an unexpected finding given the hyperglycemic and inflammatory context [84, 85]. These findings suggest that interactions among chronic hyperglycemia and pharmacological treatments may shape the microbiota composition and SCFA production [85, 86].

Our multivariable findings, showing enrichment of *Prevotella* features in individuals with T2DM aged < 60 years, align with accumulating evidence that the gut microbiota is altered in T2DM [50]. Gut dysbiosis has been consistently reported in T2DM, and differential abundance of specific microbial taxa has been implicated in metabolic dysfunction and insulin resistance [87]. *Prevotella* is a prominent member of the Bacteroidetes and has been associated with metabolic phenotypes in humans [88]. Recent metagenomic evidence indicates that colonization with *Prevotella* may increase circulating branched‐chain amino acids, which are linked to insulin resistance and metabolic risk [89]. This behavior could underlie the associations observed between *Prevotella* features and T2DM status in our cohort. Cross‐cohort shotgun metagenomic analyses involving thousands of individuals have identified consistent diabetes‐associated microbial signatures and demonstrated that several taxa display strain‐level heterogeneity linked to metabolic traits and glucose dysregulation [49]. Members of the genus *Prevotella*, particularly *Prevotella copri*, have been repeatedly associated with host metabolic phenotypes, carbohydrate metabolism, and insulin resistance pathways [90, 91]. However, their direction of association varies by dietary pattern and population structure. Multipopulation meta‐analyses show that *Prevotella* is frequently observed in metabolic disease contexts, suggesting that its presence reflects ecosystem‐level metabolic configuration rather than a uniform pathogenic role [92]. Together, current evidence supports the idea that *Prevotella* is a recurrent component of microbiota configurations associated with metabolic disease. However, their functional impact is likely context‐dependent and influenced by diet, host physiology, and strain‐level variation rather than simple presence–absence relationships.

Functional predictions further revealed a reduced GDP‐mannose biosynthesis pathway in T2DM patients aged ≥ 60 years. This pathway is essential for bacterial cell wall glycan synthesis [93]. A reduction in predicted gene abundance suggests a diminished capacity of gut bacteria to produce mannose‐rich glycoconjugates, which may influence host immune modulation [94]. Although based on in silico data, this distinctive metabolic signature may influence neuroinflammation [95]. Future validation using microglial and blood–brain barrier models is necessary to clarify GDP‐mannose’s role in gut–brain communication.

Our study provides integrative evidence that T2DM and AD may share overlapping pathogenic pathways, particularly those involving the gut‒brain axis. The gut dysbiosis observed in T2DM may promote neuroinflammation via microbial metabolites that contribute to AD pathogenesis [13].

This study has limitations that should be acknowledged. First, the cross‐sectional design limits the ability to establish causal relationships between gut microbiota composition and the clinical characteristics of T2DM and AD. Second, the relatively small sample sizes of the AD and AD–T2DM groups may limit statistical power to detect subtle microbial shifts and reduce the generalizability of the findings; however, these results provide a valuable foundation and underscore the need for future studies with larger, more representative cohorts to validate and expand upon these observations. Third, although 16S rRNA gene sequencing of the V4 region provides valuable taxonomic information, it does not allow for reliable species‐level resolution or direct assessment of functional microbial activity. Consequently, functionally distinct species within the same genus may be grouped together, and predicted functional inferences should be interpreted with caution. Future studies using shotgun metagenomics and integrated multiomics approaches will be necessary to achieve higher taxonomic and functional resolution. Shotgun metagenomics would allow more precise characterization of microbial pathways involved in SCFA biosynthesis, LPS production, carbohydrate metabolism, and host–microbe interactions relevant to metabolic and neurodegenerative processes [96]. Another limitation of this study is the absence of direct measurements of gut‐derived metabolites, such as SCFAs, circulating LPS, and inflammatory cytokines. Consequently, mechanistic interpretations are based on microbial taxonomic composition and in silico functional predictions. The use of predictive metagenomic tools such as PICRUSt2, while informative, may not accurately capture the full functional complexity of the microbiome, especially in disease states. Finally, potential confounders such as diet, formal clinical staging of AD severity, medication doses, and lifestyle factors were not controlled for or matched across groups, which may have influenced the observed microbial profiles. Future studies with longitudinal designs, larger and more balanced sample sizes, shotgun metagenomic sequencing, and more comprehensive clinical and environmental data are warranted to confirm and validate these proposed pathways.

## 5. Conclusion

This study offers novel insights into the gut microbiota patterns associated with T2DM, AD, and their comorbidity and highlights key microbial genera that may play a role in the interplay between metabolic and neurodegenerative disorders. As such, it provides a relevant contribution to a growing body of literature and a platform for more targeted, mechanistic investigations. Our study reveals a pronounced loss of microbial diversity and distinct shifts in SCFA‐producing and inflammation‐linked taxa in T2DM, AD, and AD–T2DM individuals. Additionally, predictive functional analyses revealed unique microbial metabolic alterations, including a reduced potential for GDP‐mannose biosynthesis in T2DM patients, which may affect host immune responses and neuroinflammation. Overall, these results support a role for the gut‒brain axis in the shared pathology of T2DM and AD and suggest that the gut microbiota may represent a promising target for early detection and intervention strategies in populations at risk for dementia.

## Author Contributions

Alexis M. Rodríguez‐Rosas: data curation, investigation, writing–original draft, formal analysis, and visualization. Diana L. Baldenebro‐Félix: data curation and writing–original draft. Jesús M. Peréz‐Villarreal: writing–original draft, formal analysis, and visualization. Jaime García‐Mena: data curation, methodology, and writing–original draft. Carlos A. Kawano‐Soto and Josué Camberos‐Barraza: resources and data curation. Marco A. Valdéz‐Flores: resources and validation. Loranda Calderón‐Zamora: data curation, formal analysis, and visualization. Carla E. Angulo‐Rojo: conceptualization, data curation, investigation, and visualization. Javier A. Magaña‐Gómez: supervision, writing–review and editing, conceptualization, and investigation. Alma M. Guadrón‐Llanos: writing–original draft, supervision, and writing–review and editing.

## Funding

We thank the Secretaría de Ciencia, Humanidades, Tecnología e Innovación for the financial support for the research, grants CBF2023‐2024‐3494 and CONACyT 163235 INFR‐2011‐01, and the Laboratorio de Referencia y Apoyo para la Caracterización de Genomas, Transcriptomas y Microbiomas en la Unidad Zacatenco del Cinvestav, to J.G.M.

## Ethics Statement

The studies involving humans were approved by the Research Ethics Committee of the Faculty of Medicine at the Autonomous University of Sinaloa (registration number CEI‐FM‐PI‐2022‐010). The studies were conducted in accordance with local legislation and institutional requirements. The participants provided written informed consent to participate in this study. Written informed consent was obtained from the individual(s) for the publication of any potentially identifiable images or data included in this article. Permission to use the Montreal Cognitive Assessment (MoCA) in this research was officially granted by the copyright holders for the period 2022–2026.

## Conflicts of Interest

The authors declare no conflicts of interest.

## Supporting Information

Supporting Information. This manuscript is accompanied by one Supporting file, which includes four figures and one table that complement and support the analyses presented in the main text. Additional supporting information can be found online in the Supporting Information section.

Supporting Figure 1: Rarefaction curves for each sample, grouped by clinical condition.

Supporting Figure 2: Relative abundance of microbial phyla across the different groups.

Supporting Figure 3: Shepard diagram derived from NMDS Bray–Curtis ordination.

Supporting Figure 4: Comparative analysis of predicted metabolic pathways (PICRUSt) between older controls and older individuals with T2DM.

Supporting Table 1: Significant multivariable microbial associations identified by MaAsLin2.

These materials are intended to provide additional methodological detail and strengthen the interpretation of microbiota composition and functional predictions.

## Supporting information


**Supporting Information** Additional supporting information can be found online in the Supporting Information section.

## Data Availability

The datasets generated and analyzed for this study can be found in the Alzheimer‐Diabetes Crosstalk metagenomic sequencing [PRJNA1276681 https://www.ncbi.nlm.nih.gov/bioproject/1276681].
