# Supplementary material for: Gut Microbiota Diversity and Function in Adults With Type 2 Diabetes, Alzheimer’s Disease, and Both Conditions
Source: Int J Microbiol. 2026 Apr 5;2026:5247744. doi: 10.1155/ijm/5247744 (PMC13051801; doi:10.1155/ijm/5247744)
Supplement: Supplementary file 1 — Supporting Information Additional supporting information can be found online in the Supporting Information section. [file IJM-2026-5247744-s001.zip › Supplementary_Material_Alexis.pdf]

Supplementary Material

1 Supplementary Figures

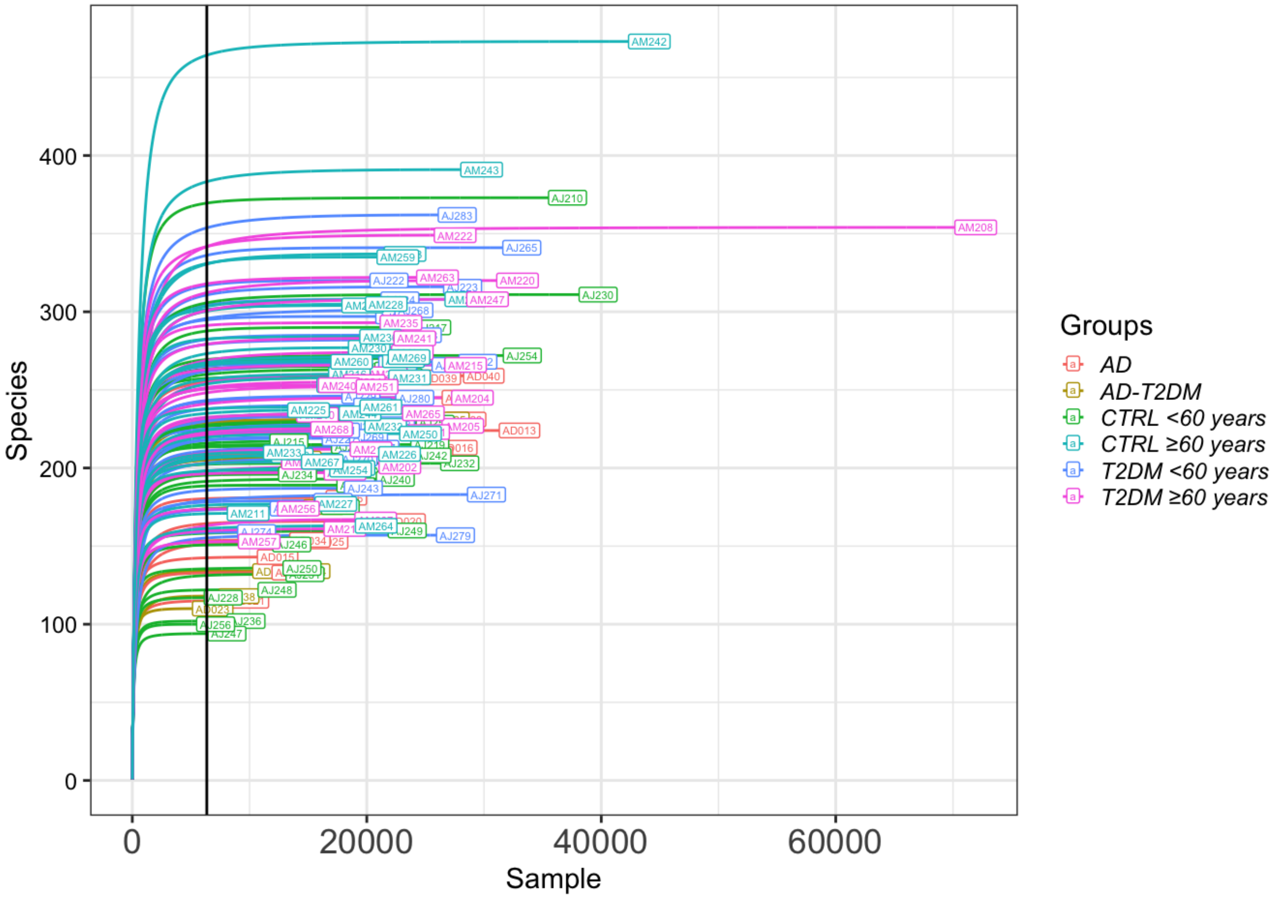

Supplementary Figure 1. Rarefaction curves for each sample, colored according to study group (AD, AD-T2DM, CTRL <60 years, CTRL ≥60 years, T2DM <60 years and T2DM ≥60 years). The horizontal axis represents the number of sequenced reads per sample, while the vertical axis indicates the number of species detected. Each line illustrates how the number of species increases as sequencing depth increases, until it approaches a plateau, suggesting adequate coverage. The black vertical line indicates the sampling depth chosen for normalization, ensuring a homogeneous comparison of microbial diversity between samples. All samples were rarefied to 6,355 reads, the depth of the sample with the fewest sequences, and rarefaction curves confirmed that this cutoff reached an asymptote.

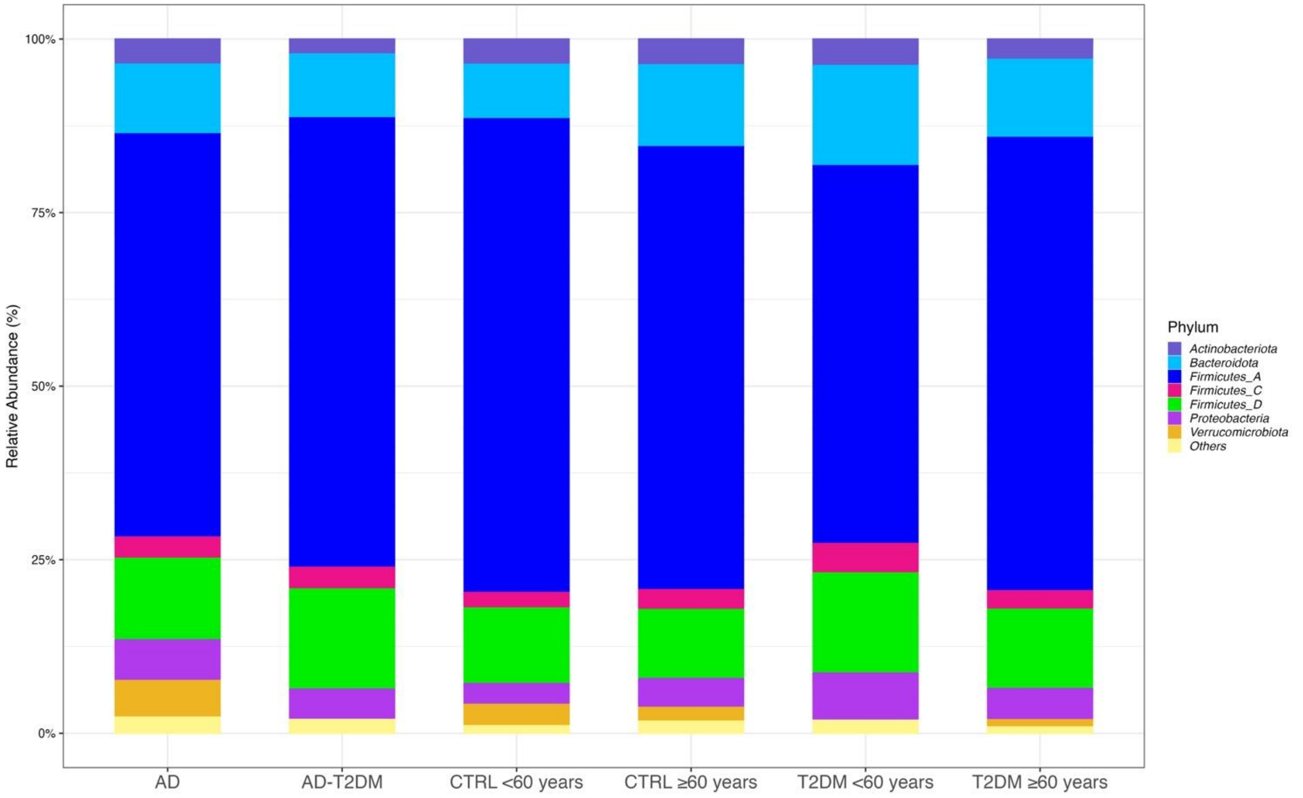

Supplementary Figure 2. Percentage distribution of microbial phyla in each study group. The stacked bars represent the relative abundance of the different phyla, concerning the variations in the overall composition of the intestinal microbiota according to the clinical condition of each group (AD, AD-T2DM, CTRL <60 years, CTRL ≥60 years, T2DM <60 years and T2DM ≥60 years).

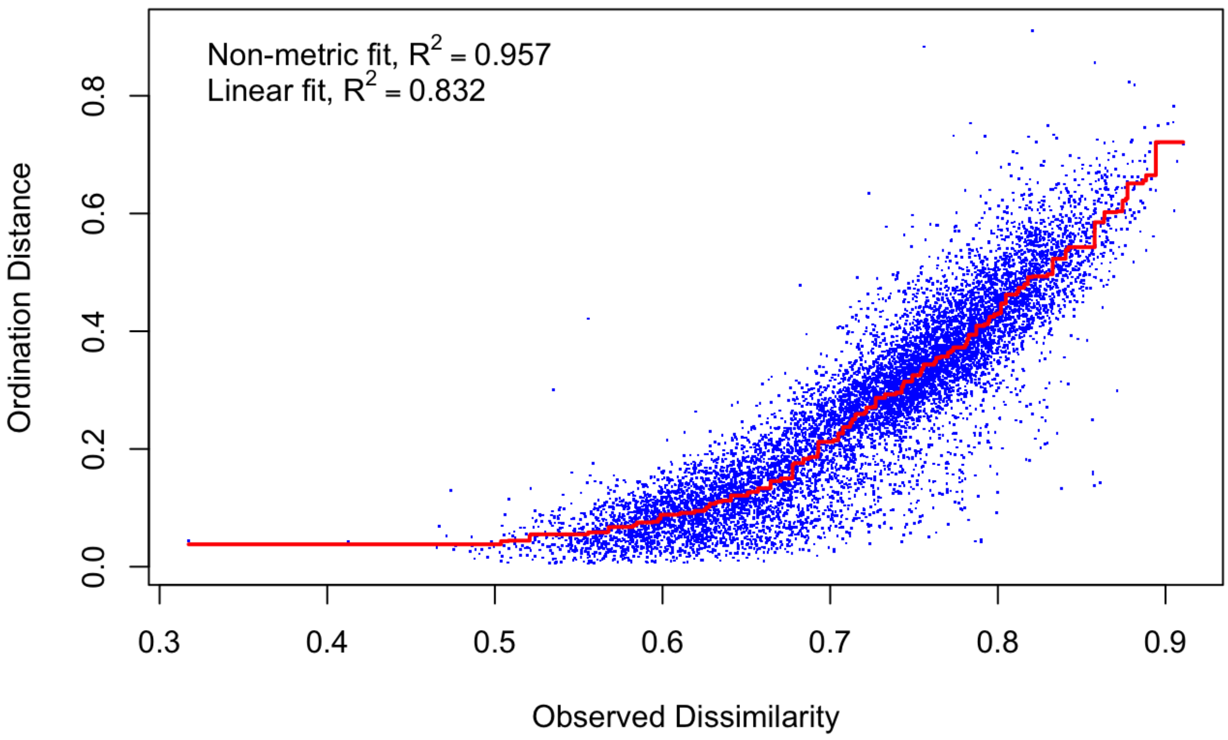

Supplementary Figure 3. Shepard diagram for NMDS Bray–Curtis ordination. The non-parametric fit reaches a value of R^2=0.964, while the linear fit presents an R^2=0.88, indicating a high agreement between the original distances and the configuration obtained in the NMDS.

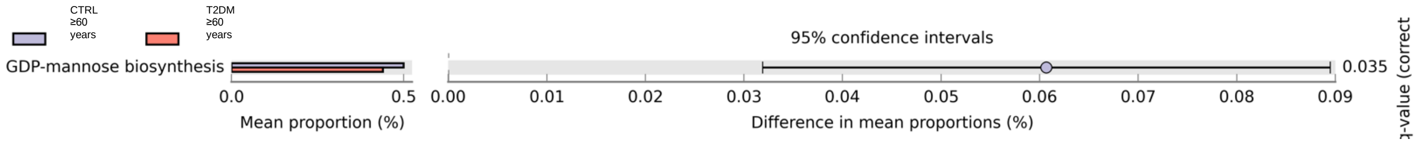

Supplementary Figure 4. Comparison of predicted metabolic pathways (PICRUSt pathway) between CTRL ≥60 years vs T2DM ≥60 years). On the left is the mean proportion of each pathway in both groups, where violet corresponds to CTRL ≥60 years and red to T2DM ≥60 years. On the right is the difference in the mean proportions, together with the 95% confidence interval and the q value (adjusted p value). All pathways depicted exhibit statistically significant differences.
